# The Incidence Rate of Esophageal Cancer in Saudi Arabia: An Observational and a Descriptive Epidemiological Analyses

**DOI:** 10.3389/fpubh.2022.818691

**Published:** 2022-03-23

**Authors:** Ahmad Almatroudi

**Affiliations:** Department of Medical Laboratories, College of Applied Medical Sciences, Qassim University, Buraydah, Saudi Arabia

**Keywords:** esophageal cancer, age-standardized incidence rates, crude incidence rates, Saudi Arabia, epidemiology

## Abstract

**Introduction:**

Esophageal cancer ranks the sixth most diagnosed cancer worldwide, and the morality incidence of this disease is rapidly growing worldwide. A retrospective observational population-based epidemiological study of esophageal cancer has been conducted, and data are based on the cancer registry of the National Health Information Center Saudi from 2006 to 2016. This study described the age-standardized incidence rates (ASIRs) and crude incidence rates (CIRs) of esophageal cancer based on age groups, diagnosis year, and administrative areas in Saudi Arabia populations to examine its distributions and trends in Saudi Arabia.

**Method:**

For the statistical assessment of data, sex ratio, *t*-test, the Kruskal–Wallis test, and descriptive statistics were performed using SPSS version 20.0 (IBM Corporation, Armonk, NY, USA). A total of 755 and 597 cases of esophageal cancer in men and women, respectively, were reported from 2006 to 2016 in Saudi Arabia.

**Results:**

Out of all esophageal cases, the highest number of cases was observed in the age group <75 years among both men and women, whereas the lowest percentage and mean number of esophageal cancer cases among men and women were reported in the younger age group between 0 and 29 years. Within the geographical regions, Tabuk and Qassim regions recorded the highest mean CIR and ASIR among men. In the Northern region of Saudi Arabia, the maximum CIR and ASIR sex ratio was observed, whereas minimum mean CIR and ASIR were reported in Jouf and Jazan regions, respectively, among men. Madinah and Tabuk regions had the maximum mean CIR and ASIR, respectively, among women for esophageal cancer. The Northern region recorded minimum mean CIR and ASIR among women.

**Conclusion:**

Maximum substantial changes of ASIRs for esophageal cancer in men and women from 2006 to 2016 were found in the Tabuk region, while Jazan and Northern regions exhibited least substantial changes of ASIRs in men and women, respectively.

## Introduction

Cancer is a well-known cause of death globally and is a huge impediment in improving the human life expectancy around the globe. According to the 2019 World Health Organization (WHO) report, cancer is the first or second prominent cause of death among 112 countries out of 183 countries, and it is classified as third or fourth in other 23 countries (1). The International Agency for Research on Cancer (IARC) projected that cancer burden has grown to around 18 million new cases with 9 million death occurrences in 2018 alone (1).

Esophageal carcinoma ranks as the eighth most common cancer in the world and the sixth most leading cause of cancer-related deaths (Table 1) (2). Esophageal cancer is characterized by inferior prognosis during the diagnosis along with a high rate of mortality with the 5-year survival rate of <25%. It is also complicated due to considerable differences in its histopathology, mortality, and incidence rate based on a geographical area (3). Adenocarcinoma and squamous cell carcinoma are the two most common types of esophageal cancer (4). According to the Surveillance, Epidemiology, and End Results (SEER) cancer statistics 2021, around 19,260 new cases of esophageal cancer and approximately 15,530 deaths worldwide, which respectively accounted for 1% of all new cancer cases and for 2.6% of all cancer deaths, were reported (5). Among gastric cancers, esophageal cancer stands at the fourth place with 6.3 age-standardized incidence rate (ASIR), whereas colorectal cancer is the topmost gastric cancer with the highest ASIR (19.7) (Table 1) (2). Totally, the 5-year survival rate of all types of esophageal cancer persists poorly at around 20% (6). Despite the poor prognosis of esophageal cancer, significant advances in the treatment of cancer have resulted in cancer mortality in the last 40 years. Between the two common esophageal cancer types, adenocarcinoma has a better prognosis in comparison to squamous cell carcinoma (7). A study examined the survival trends of esophageal adenocarcinoma and squamous cell carcinoma from mid-1970s to late-1990s. This study reported that the 5-year relative survival rate of esophageal adenocarcinoma and squamous cell carcinoma increased from 5.7 to 13.6 and from 4.5 to 11.8, respectively, during the period of study (8).

**Table 1:** Age-standardized mortality rate (ASMR), age-standardized incidence rates (ASIR), crude rates, and numbers of few top cancer cases across the globe (2).

**Cancer**	**Mortality**			**Incidence**		
	**ASMR**	**Crude rate**	**Number**	**ASIR**	**Crude rate**	**Number**
All cancers	101.1	125.2	9555027	197.9	236.9	18078957
Breast cancer	13.9	16.6	626679	46.3	55.2	2088849
Lung cancer	18.6	23.1	1761007	22.5	27	2093876
Colorectal cancer	8.9	11.5	880792	19.7	24.2	1849518
Stomach	8.2	10.3	782685	11.1	13.5	1033701
Liver	8.5	10.2	781631	9.3	11	841080
Esophagus	5.5	6.7	508585	6.3	7.5	572034
Pancreas	4.4	5.7	432242	4.8	6	458918

*ASIR, age-standardized incidence rate; ASMR, age-standardized mortality rate*.

In Saudi Arabia, ASIR of all types of cancer is 88.7 per 1,00,000 individuals whereas age-standardized mortality rate (ASMR) is 43.3 per 1,00,000 individuals (9). According to Globocan's (2020) report, within the gulf regions, the highest number of new cancer cases and deaths due to esophageal cancer in men was found in Saudi Arabia followed by Iraq, whereas the highest number of new cancer cases and deaths in women due to esophageal cancer was reported in Iraq followed by Saudi Arabia in all the age groups (Table 2) (10).

**Table 2:** Estimated number of new cases and deaths (per 1,00,000 individuals) in 2020 in both sexes, men, and women among the gulf (10).

**Country**	**Both sexes**		**Men**		**Women**	
	**New cases**	**Deaths**	**New cases**	**Deaths**	**New cases**	**Deaths**
Iraq	254	240	126	117	128	123
Saudi Arabia	246	233	154	145	92	88
Oman	35	33	23	22	12	11
UAE	30	27	22	20	8	7
Kuwait	28	27	21	20	7	7
Qatar	17	15	11	10	6	5
Bahrain	14	13	9	8	5	5

Dietary habits, nutritional regime, and life style conditions have also been linked to esophageal cancer. Increased physical exercise and a higher intake of fruits and vegetables are associated with a low risk of esophageal cancer while the consumption of pickled vegetables is associated with a high risk of esophageal cancer (11), (12). The consumption of alcohol, smoking, and a low intake of vegetables and fruits were accounted for 89% of risk factors of esophageal cancer in the USA (11). In this paper, the aim is to study the epidemiologic parameters of ASIRs and crude incidence rates (CIRs) of esophageal cancer by age group, diagnosis year, and administrative areas in Saudi Arabia populations to examine its distribution and trends in Saudi Arabia. To achieve this aim, an observational and detailed epidemiologic study of esophageal cancer case distribution and prevalence based on the cancer registry of Saudi Arabia from 2006 to 2016 has been carried out.

## Materials and Methods

A retrospective observational population-based epidemiological study of esophageal cancer has been conducted based on the 10-year cancer incidence report from January 2006 to December 2016 published by National Health Information Center, Ministry of Health, Saudi Arabia in 2020 (13). The data of esophageal cancer diagnosed cases for the entire Saudi Arabia between 2006 and 2016 have been accessed *via* open access public domain, available through the SCR annual report; hence, no ethical approval is obligatory for this study. The Saudi cancer registry data provides a comprehensive report on the incidence rate of cancer, ASIR, and CIR for all the thirteen administrative regions of Saudi Arabia, stratified on the basis of the year of diagnosis and demographic characteristics for the period of 10 years between 2006 and 2016. The present study includes incidences of esophageal cancer only among Saudi Arabia residents and does not include non-Saudi residents. The report thoroughly present most common types of cancer by gender in each of the thirteen administrative regions of Saudi Arabia based on the consolidated cancer data from all the administrative regions.

The statistical analysis was conducted using SPSS version 20.0 (IBM Corporation, Armonk, NY, USA). ASIRs and CIRs of 2006–2016 were reported from Saudi cancer registry reports, and the differences between ASIRs and CIRs were determined to analyze the esophageal cancer trend among female and male populations in the different administrative regions of Saudi Arabia. The comparison between ASIRs and CIRs of esophageal cancer cases among men and women was accomplished using an independent sample *t*-test. A nonparametric Kruskal–Wallis *H*-test was performed to correlate the CIRs and the ASIRs of esophageal cancer present in the different administrative regions of Saudi Arabia. The male–female ratio of esophageal cancer was computed from ASIR, CIR categorized by the year of diagnosis, sex, administrative areas, and age groups. A 5-year class interval has been set to categorize the population data into 16 standardized age groups: 0–4 years old, 5–9 years old, 10–14 years old, 15–19 years old, 20–24 years old, 25–29 years old, 30–34 years old, 35–39 years old, 40–44 years old, 45–49 years old, 50–54 years old, 55–59 years old, 60–64 years old, 65 year old, 69 year old, 70–74 years old, and 75 year old and above for this study.

## Results

### Esophageal Cancer Among Men

**Figure 1:**
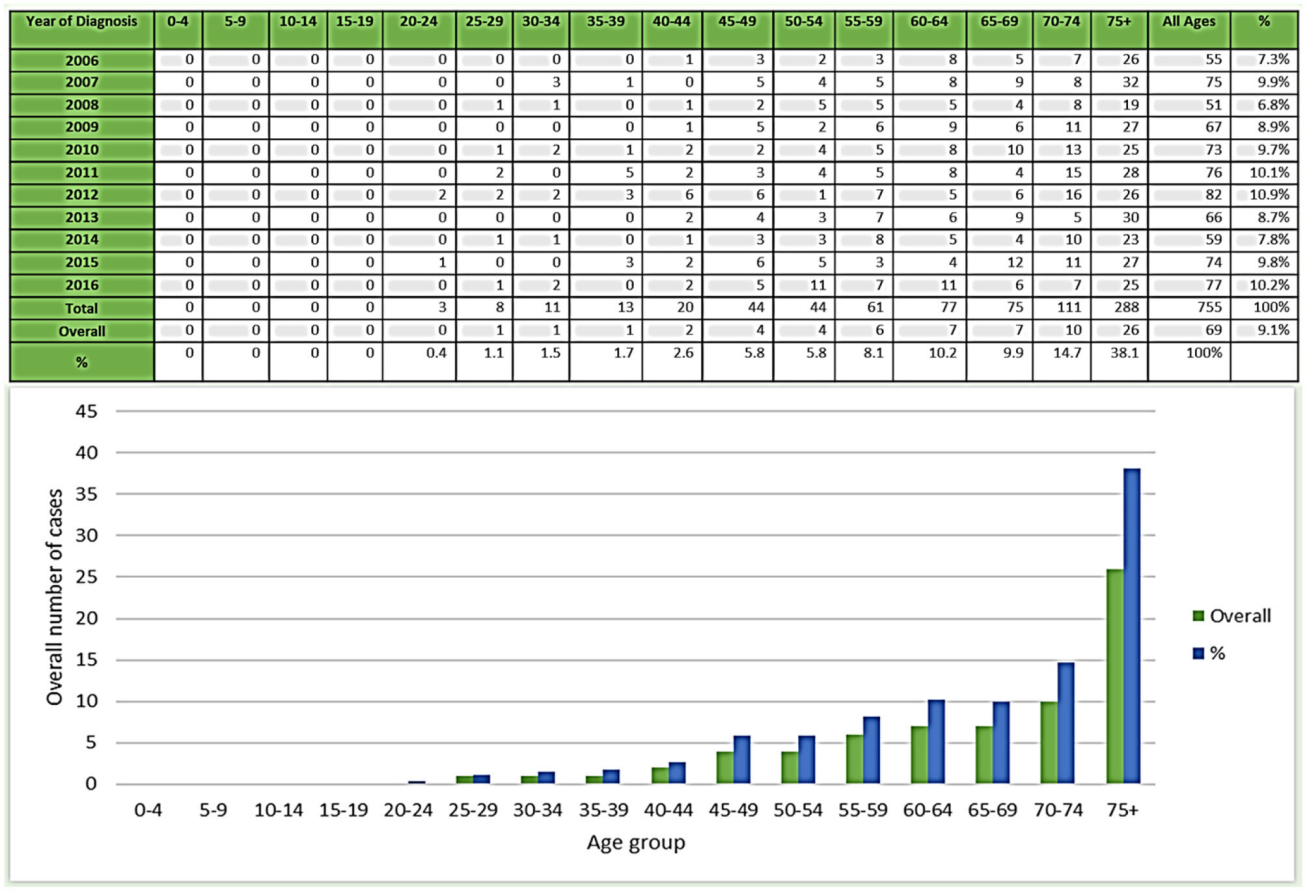
Number and percentage of esophageal cancer cases in men during 2006–2016.

A total of 755 cases of esophageal cancer among men were reported from 2006 to 2016 in Saudi Arabia (13). The incidence rate of esophageal cancer cases increased slightly between 2006 and 2016 (Table 3). The number of cases pertaining to esophageal cancer increased from 55 in 2006 to 75 in 2016 (Figure 1). In the time span of these 10 years, the highest number of esophageal cancer cases was recorded in 2012 among men (82). The percentage and mean number of esophageal cancer cases reported in Saudi Arabia categorized by age group have also been computed. The class-width of age groups was kept at 5 years, starting from 0 to 4, 5 to 9, 70 to 74 years, and to the last age group of 75 years and above. While the percentage of esophageal cancer cases among men per year from 2006 to 2016 was 9.1%, the mean number of esophageal cancer cases was 69. Available data highlight that, from 2006 to 2016, esophageal cancer cases were mostly diagnosed in men belonging to the age group of 75 years and above. Out of all 755 cases, the highest number of cases were primarily found to be concentrated in the age group of 75 years and above with a total of 288 cases, followed by the 70–74 age group with 111 cases.

**Table 3:** The difference between age-standardized incidence rate (ASIR) and crude incidence rates (CIR) of esophageal cancer between 2016 and 2006.

**Area**	**Sex**	**ASIR/CIR**	**2016**	**2006**	**Difference**
Riyadh	Male	ASIR	1.6	1.1	0.5
		CIR	1	0.5	0.5
	Female	ASIR	0.5	1	−0.5
		CIR	0.4	0.5	−0.1
Makkah	Male	ASIR	1.1	1.6	−0.5
		CIR	0.9	1	−0.1
	Female	ASIR	0.9	1.5	−0.6
		CIR	0.7	0.9	−0.2
Eastern Province	Male	ASIR	0.6	1.2	−0.6
		CIR	0.4	0.4	0
	Female	ASIR	0.6	2.2	−1.6
		CIR	0.4	0.9	−0.5
Jouf	Male	ASIR	0	2.3	−2.3
		CIR	0	1.2	−1.2
	Female	ASIR	0	0	0
		CIR	0	0	0
Tabuk	Male	ASIR	4.2	1.8	2.4
		CIR	2.2	0.6	1.6
	Female	ASIR	1.9	3.1	−1.2
		CIR	0.9	1	−0.1
Northern Region	Male	ASIR	2.1	0	2.1
		CIR	1.4	0	1.4
	Female	ASIR	0.7	0	0.7
		CIR	0.7	0	0.7
Hail	Male	ASIR	0.6	0.6	0
		CIR	0.4	0.4	0
	Female	ASIR	0	0	0
		CIR	0	0	0
Qassim	Male	ASIR	1	1.8	−0.8
		CIR	0.8	1.2	−0.4
	Female	ASIR	0.6	1.7	−1.1
		CIR	0.4	1.9	−1.5
Najran	Male	ASIR	0.9	1.1	−0.2
		CIR	0.5	0.5	0
	Female	ASIR	0.7	0	0.7
		CIR	0.5	0	0.5
Asir	Male	ASIR	0.5	0.5	0
		CIR	0.6	0.4	0.2
	Female	ASIR	0.4	0.2	0.2
		CIR	0.5	0.3	0.2
Baha	Male	ASIR	2.2	2.4	−0.2
		CIR	2.8	2.5	0.3
	Female	ASIR	0	0	0
		CIR	0	0	0
Jazan	Male	ASIR	0.2	0.5	−0.3
		CIR	0.2	0.4	−0.2
	Female	ASIR	0.6	1.6	−1
		CIR	0.5	0.7	−0.2
Madinah	Male	ASIR	0.3	0.3	0
		CIR	0.1	0.2	−0.1
	Female	ASIR	1.2	3.7	−2.5
		CIR	0.7	2.7	−2

Therefore, an increasing tendency of esophageal cancer cases can be associated with an increase in age as the cancer incidences can be observed to increase with age, particularly after 45 years and above. While the lower percentage and mean number of esophageal cancer cases can be observed in lower age groups with declining cases in the age groups of 45 and below. A declining trend in the number of cancer incidences can be observed in the age groups of 45 and less. The lowest incidences have been reported for the younger age groups between 0 and 29 years (Figure 1).

#### Esophageal Cancer: CIRs Among Men Between 2006 and 2016 in the Region

**Figure 2:**
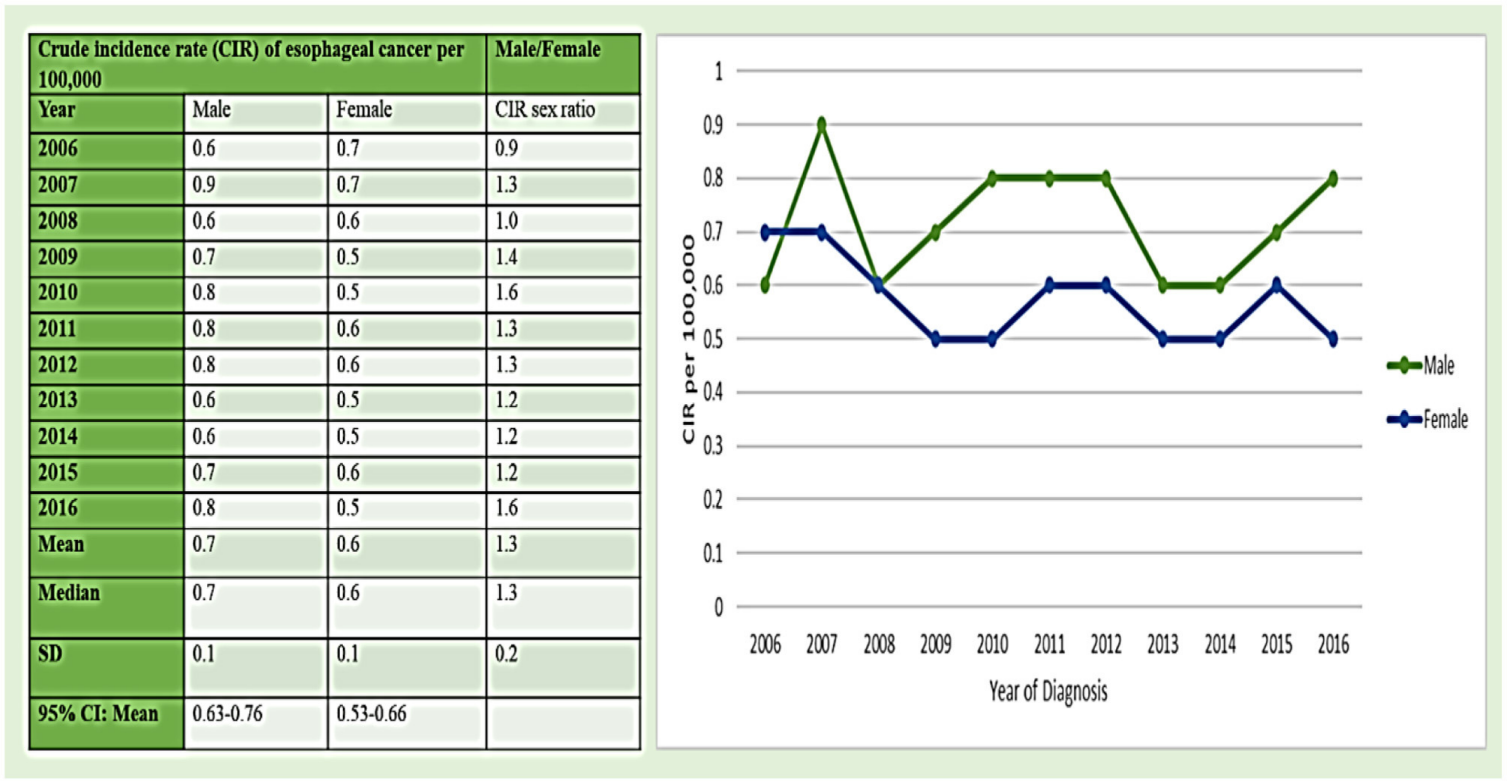
The crude incidence rate (CIR) of esophageal cancer cases per 1,00,000 between men and women from 2006 to 2016.

The CIRs of *esophageal cancer* in Saudi men between 2006 and 2016 time period per 100,000 men reflect a mixed picture with increasing and decreasing trends. While it was 0.6/1,00,000 in 2006, it increased to 0.9 in the mere time period of 1 year, i.e., in 2007, and then again decreased to again 0.6 in 2008 thereafter reflecting an increasing trend from 2008 to 2012. Further, the CIR reduced during 2013 and 2014 and then again reflect an increasing trend during 2015 and 2016 (Figure 2). The maximum CIR was observed in 2007 with a value of 0.9/1,00,000, followed by 2010, 2011, 2012, and 2016 with 0.8 CIR. The mean of CIR sex ratio (male/female) was 1.3 for the period between 2006 and 2016 (Figure 2).

**Figure 3:**
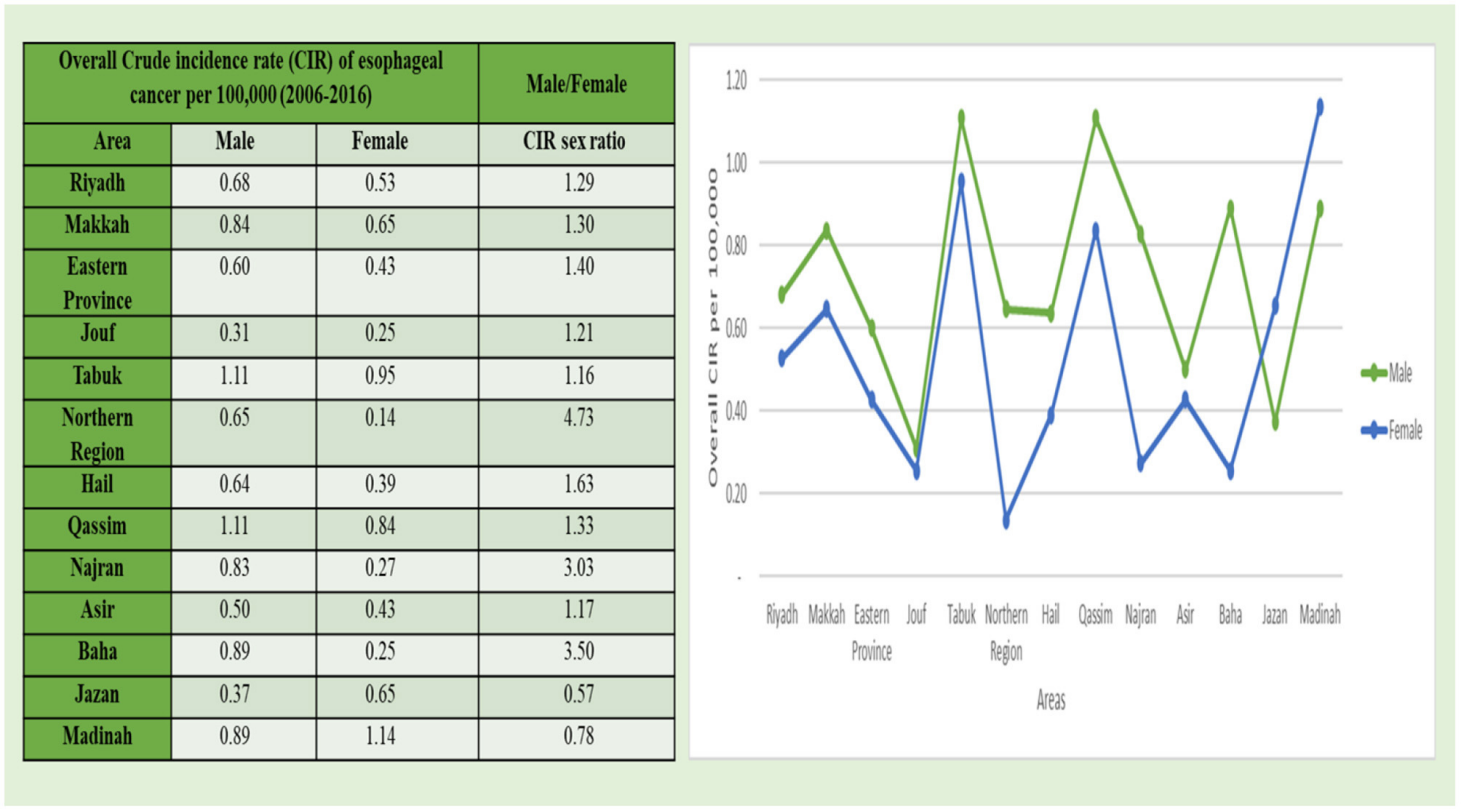
The overall CIR of esophageal cancer cases per 1,00,000 between men and women according to administrative areas during 2006–2016.

The mean CIRs of esophageal cancer in men were also classified based on the thirteen administrative areas of Saudi Arabia as categorized in the cancer registry of Saudi Arabia (Figure 3). The Northwestern Tabuk region and the western central Qassim areas had the highest mean CIR of 1.11/1,00,000 among men, followed by Baha and Madinah areas with 0.89/100,000 incidence rate. The Kruskal–Wallis *H*-test was done on overall CIRs for all areas from 2006 to 2016 in men because the data were not ensuing a normal-distribution and observed statistical significance for these areas in comparison to other administrative areas. Furthermore, in the Northern region of Saudi Arabia, the maximum CIR sex ratio was observed with the 4.73/1,00,000 rate, followed by the Baha region at 3.50/1,00,000 incidence rate for esophageal cancer, and the minimum mean CIR was reported in the Jouf region with 0.31/1,00,000 incidence rate among men (Figure 3).

#### Esophageal Cancer: ASIRs Among Men During 2006–2016 in the Region

**Figure 4:**
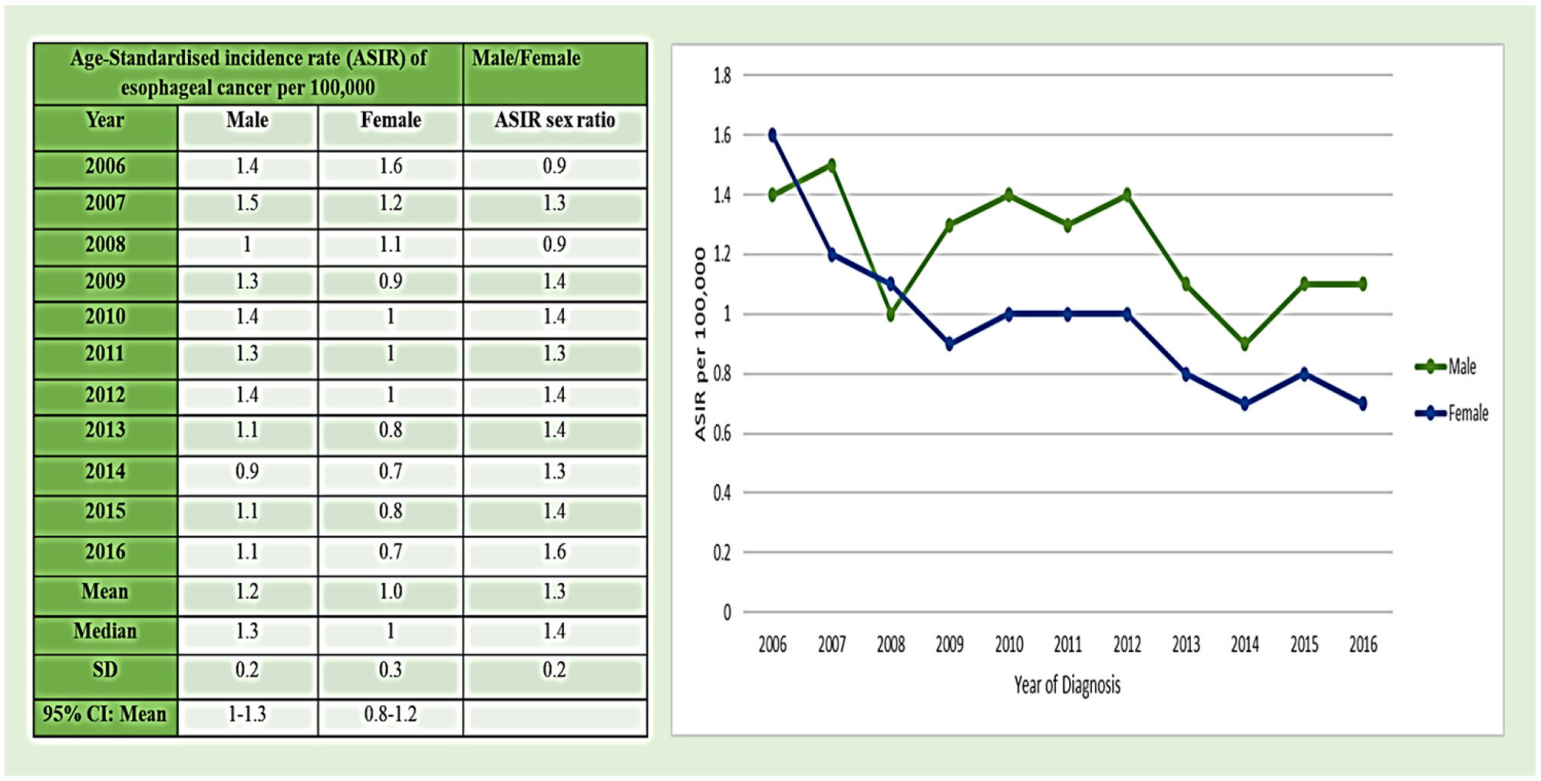
Age-standardized incidence rate (ASIR) of esophageal cancer cases per 1,00,000 between men and women during 2006–2016.

The ASIRs of esophageal cancer in men were reported from the cancer registry of Saudi Arabia from 2006 to 2016 per 100,000 men (Figure 4). The value of ASIRs of esophageal cancer shows a dynamic trend with increasing and decreasing ASIRs from 2006 to 2016 in Saudi Arabia. The maximum value of ASIR was reported in 2007 with 1.5/1,00,000 incidence rate among men and in 2006, 2010, and 2012 with 1.4/1,00,000 incidence rate. The lowest ASIR value was recorded in 2014 with 0.9/1,00,000 incidence rate among men. The minimum value of ASIR was observed in 2014 with 0.9/1,00,000 incidence rate. Moreover, the mean ± standard deviation (SD) of ASIR was 1.2 ± 0.2 among men in esophageal cancer. The mean ± SD of ASIR sex ratio from 2006 to 2016 was 1.3 ± 0.2 per 1,00,000 individuals (both male and female) but it was higher in men with 1.2/1,00,000 as compared to women (Figure 4).

**Figure 5:**
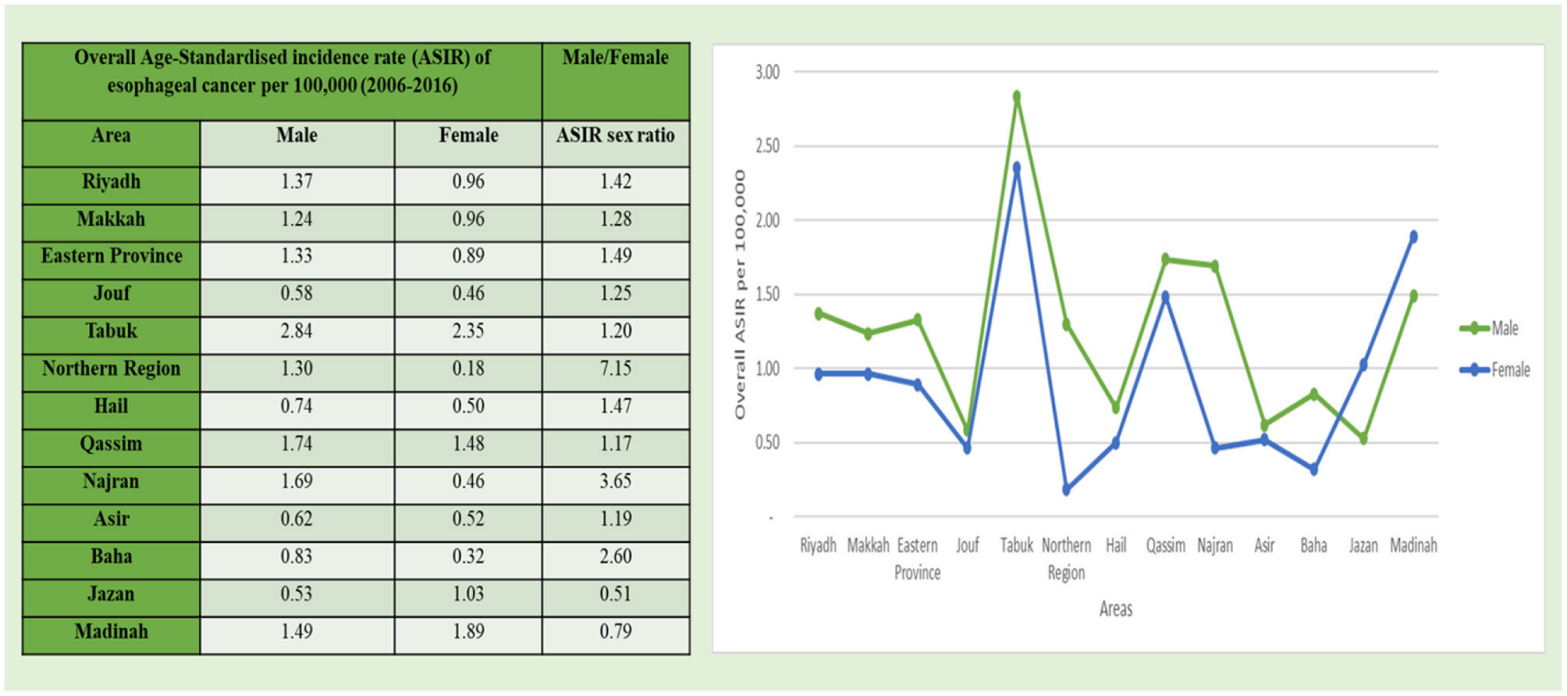
Overall ASIR of esophageal cancer cases per 1,00,000 between men and women according to administrative areas during 2006–2016.

By geophysical analysis of the extent and distribution of the mean ASIR values of esophageal cancer among men, the data were also classified by the thirteen administrative areas of Saudi Arabia as categorized in the cancer registry of Saudi Arabia (Figure 5). Among all the 13 administrative divisions, the Tabuk region with 2.84/1,00,000 incidence rate had the highest mean ASIR, followed by the Qassim region with 1.74/100,000 incidence rate and the Najran region with 1.69/1,00,000 incidence rate. The Kruskal–Wallis *H*-test was conducted on overall ASIRs for all areas from 2006 to 2016 for men because the data were not ensuing a normal-distribution, and a statistical significance for these areas was observed in comparison to other administrative areas. Furthermore, the Northern region of Saudi Arabia reflected a maximum ASIR sex ratio with 7.15/1,00,000 incidence rate, followed by the Najran region with 3.65/1,00,00 rate for esophageal cancer and the minimum mean ASIR was reported in the southern most region of Saudi Arabia, i.e., in the Jazan region with 0.53/1,00,000 incidence rate among men (Figure 5).

### Esophageal Cancer Among Women

**Figure 6:**
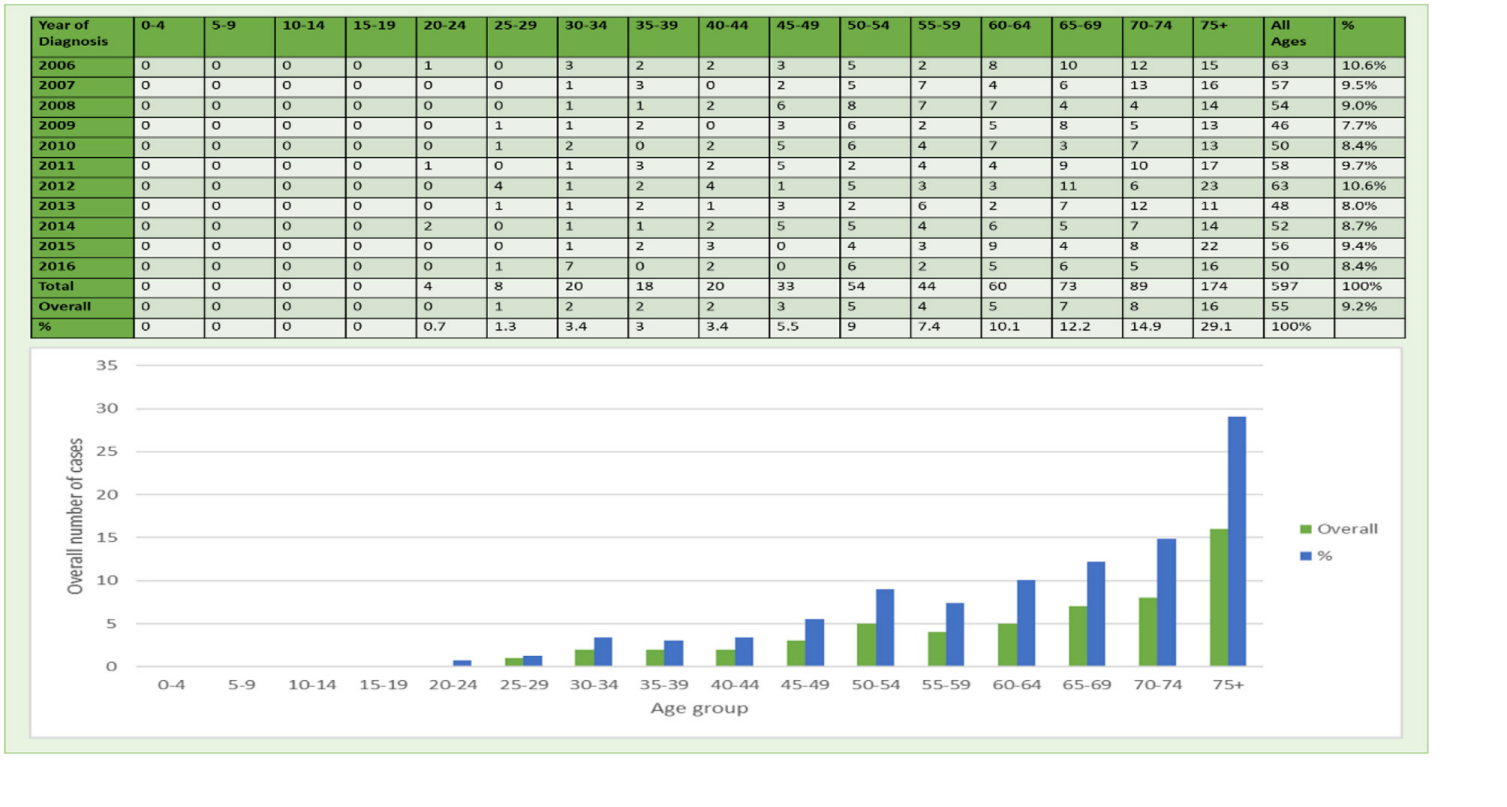
Number and percentage of esophageal cancers in women during 2006–2016.

A total of 597 cases of incidences of esophageal cancer among women were reported from 2006 to 2016 in Saudi Arabia (13). The incidence of the number of esophageal cancer cases reflects a declining trend slightly between 2006 and 2016 (Table 3). While there were 63 cases in 2016, which decreased to 50 cases of esophageal cancer in 2016 (Figure 6). According to the Saudi cancer registry, the highest number of esophageal cancer cases in women (63) were found in 2006 and 2012 between 2006 and 2016. The percentage and mean number of esophageal cancer cases reported in Saudi Arabia categorized by age group were also computed. The class-width of age groups was kept at 5 years, starting from 0 to 4, 5 to 9, 70 to 74 years and to the last age group of more than 75 years. The percentage of esophageal cancer cases in women per year from 2006 to 2016 was 9.2% and mean esophageal cancer cases were 55. From 2006 to 2016, the esophageal cancer cases among women were mostly diagnosed in women belonging to the age group of more than 75 years. Out of all 597 cases, the highest number of cases were only found in the age group of >75 years with 174 cases, accounting for the highest share percentage cases, i.e., (29% of all cases) among all the age groups in women, followed by the 70–74 age group with 89 cases and 14% of total number of cases in women. Therefore, an increasing tendency of esophageal cancer cases can be associated with increasing age as the cancer incidences can be observed to be increasing with age, particularly after the age group of 45 years and above in women. Meanwhile, lower percentage and mean number of esophageal cancer cases can be observed in lower age groups with declining cases in the age group of 45 and below. A declining trend in the number of cancer incidences can be observed in the age group of 45 and less. The lowest incidence rate has been reported for the younger age groups between 0 and 29 years. The lowest percentage and mean number of esophageal cancer cases were reported in the younger age groups (0–19 year age) among women (Figure 6).

#### Esophageal Cancer: CIRs Among Women 2006–2016 in the Region

The CIRs of esophageal cancer cases in women in Saudi Arabia showcase a declining trend of esophageal cancer cases from 2006 to 2016 (Figure 2). The maximum CIR was observed in 2006 and 2007 with 0.7/1,00,000 incidence rate followed by at 0.6/1,00,000 incidence rate in 2008, 2011, 2012, and 2015, respectively. The mean ± SD of CIRs from 2006 to 2016 in women were 0.6 ± 0.1 (male/female) (Figure 2).

The mean CIRs of esophageal cancer in women were also analyzed based on the geographical distribution and extent based on the thirteen administrative areas of Saudi Arabia as categorized in the cancer registry of Saudi Arabia (Figure 3). The western administrative region of Madinah with 1.14/1,00,000 incidence rate had the highest mean CIR among women, followed by Tabuk region with 0.95/1,00,000 cases and Qassim region with 0.84/100,000. The Kruskal–Wallis *H*-test was conducted on overall CIRs for all areas from 2006 to 2016 for female cases because the data were not ensuing a normal-distribution, and a statistical significance for these areas was observed in comparison to other administrative areas. Furthermore, the minimum mean CIR was reported in the Northern region with 0.14/1,00,000 incidence rate among women followed by the Southern region of Najran region with 0.46/1,00,000 incidence rate (Figure 3).

#### Esophageal Cancer: ASIRs Among Women 2006–2016 in the Region

Age-standardized incidence rates of esophageal cancer in women were also analyzed based on the cancer registry of Saudi Arabia from 2006 to 2016 year per 100,000 women (Figure 4). The value of ASIRs of esophageal cancer reflects a gradual decline in the trend from 2006 to 2016 in Saudi Arabia. The maximum value of ASIR was reported in 2006 with 1.6/1,00,000 incidence rate, followed by 2007 with 1.2/100,000 incidence rate. The minimum value of ASIR was observed in 2014 and 2016 with 0.7/1,00,000 incidence rate. Moreover, the mean ± SD of ASIR was 1.0 ± 0.3 among women in esophageal cancer (Figure 4).

Geo-physically, analyzing the extent and distribution of the mean ASIR values of esophageal cancer in women was also classified based on the thirteen administrative divisional areas of Saudi Arabia as categorized in the cancer registry of Saudi Arabia (Figure 5). The North Western Tabuk region with 2.35/1,00,000 incidence rate had the highest mean ASIR, followed by the Madinah region with 1.89/1,00,000 incidence rate (also located in the Northwestern region) followed by the Qassim region with 1.48/1,00,000 incidence rate. The Kruskal–Wallis *H*-test was done on overall ASIRs for all areas from 2006 to 2016 in women because the data were not ensuing a normal-distribution, and a statistical significance for these areas was observed in comparison to other administrative areas. Furthermore, the minimum mean ASIR was reported in the Northern region with 0.18/1,00,000 incidence rate of esophageal cancer among women (Figure 5).

## Discussion

A comprehensive assessment of esophageal cancer cases pertaining to all administrative areas of Saudi Arabia and including CIRs and ASIRs of esophageal cancer cases is important for a better understanding of the baseline extent and distribution of cancer prevalence in the Saudi Arabia population. In the current study, we evaluated CIR and ASIR trends of esophageal cancer. This study includes a comprehensive analysis of the distribution of esophageal cancer between men and women pertaining to 16 standardized age groups and in various administrative areas of Saudi Arabia. The present study is the first detailed analysis of esophageal cancer among men and women in various Saudi Arabia regions based on the database of PubMed. The study also examines year wise trends and patterns of the prevalence of esophageal cancer in all the thirteen administrative divisions of Saudi Arabia areas and explored the significance of disease among the population.

Different geographic regions indicate the role of environmental factors in the etiology of esophageal cancer. For instance, in nations like Switzerland and France, a high frequency of esophageal cancer is due to excessive smoking and alcohol intake (14). In this study, the maximum mean ASIR for esophageal cancer in men and in women from 2006 to 2016 was found in the Northwestern region of Tabuk. This suggests that the population of Tabuk area was highly vulnerable to the esophageal cancer risk as compared to other areas. Furthermore, the impact of lifestyle, environment, and genetic factors might be related with an increase in the ASIR of esophageal cancer in both men and women in the population of Tabuk. Thus, a detailed epidemiologic study is required to identify the key risk factors linked with the rise of ASIR of esophageal cancer in the Tabuk region of Saudi Arabia. Existence of industrial units might also be considered as one of the contributing factors for escalated incidences of cancer in the Tabuk region (15).

Feldman et al.'s (14) study suggested that the deficiency of vitamin D increases the risk of development of cancer and that taking vitamin D supplements could be a reliable and an efficient approach to decrease the incidence of cancer (16). Around 84% of the total population of Saudi Arabia has vitamin D deficiency (17). Moreover, severe vitamin D deficiency was observed in all students at King Faisal University, Saudi Arabia (18). It was also observed that, during 2006–2016, the Jazan and Northern regions had the minimum mean ASIRs for esophageal cancer in men and women, respectively. The low incidence rate might be due to a variation in environmental factors or other factors in the regions with lower ASIRs as compared with the regions with higher ASIRs. With fewer adaptations of western lifestyle or few industries, poor healthcare facilities and availability could be the reasons for a low incidence rate in these areas or a low recording of cancer cases in comparison to the regions with high incidence rates. Obesity can also be linked to higher esophageal cancer cases, for instance, the Riyadh region of Saudi Arabia exhibited 35% prevalence of obesity, which also had a high incidence rate of esophageal cancer in men and women in comparison with the Jazan region, which exhibited only 2% prevalence of obesity (19). Hence, it is required to study environmental, lifestyle, and genetic factors in esophageal cancer for a better understanding of disorder and good prognosis and treatment.

Substantial variations were also found in the CIRs of esophageal cancer among men as well as women in various administrative areas during 2006–2016 (Figure 3). The maximum CIR was observed in the North Western Tabuk and the Western central Qassim regions with 1.11/1,00,000 incidence rate among men, whereas in women, the maximum CIR was reported in Madinah with 1.14/1,00,000 incidence rate. The minimum substantial change in CIR was observed in the Southern most Jouf region and the Northern regions among men and women, respectively. In men, the maximum ASIR as well as the maximum CIR were observed in the Tabuk region. This highest CIR and ASIR in the Tabuk region among men indicate that this region is the front runner in the major substantial changes of CIRs and ASIRs for esophageal cancer from 2006 to 2016. In women, the minimum ASIR and the minimum CIR were reported in the Northern region.

Certain factors and lifestyle conditions have also been linked with the global cancer incidence rate. Less consumption of alcohol, smoking cessation, an intake of fruits and vegetables rich in antioxidants, and exercise can reduce the risk of development of esophageal cancer (20). Coffee and tea have been widely examined as the potential risk factors linked with esophageal cancer. Foods rich in nitrogenous compounds are usually associated with a high incidence of esophageal cancer in some Chinese regions (3). A meta-analysis study observed the odds ratio of 2 for people who take meal rich in nitrogenous components in comparison with individuals who do not (21). Awareness of disease, healthy lifestyle, and a reduction in age for the screening of esophageal cancer can help in early prognosis and diagnosis of disease and also can be a potential approach in reducing the incidence rate in the young and adult individuals of population, which may trouble the healthcare system of a country in the following decades (3, 21, 22). Prevailing reluctance among women for diagnosis in the past but increasing awareness and access to medical facility especially among women in Saudi Arabia are also important for cancer prevention in the region, which is gradually improving (23). National and regional programs, strategies, and multilevel diagnosis are required to reduce the rate of morbidity, mortality, and incidence of esophageal cancer (24, 25) along with nutritional regime and lifestyle factors (26).

Globocan's (2020) report places esophageal cancer at the eighth position in the number of new cases in 2020 across the globe and at the sixth position in the number of deaths in 2020 with 5,44,076 deaths worldwide (1, 10). The current study is a step in highlighting and describing the trend of esophageal cancer among Saudi Arabian population from 2006 to 2016. The study provides the national projection of ASIR and CIR of esophageal cancer in the different regions of Saudi Arabia. The assets of the present study involve the utilization of esophageal cancer incidence data from a genuine registered and published source, i.e., Saudi cancer registry. However, the availability of the last published data from 2006 to 2016 on annual bases in the Saudi cancer registry restricts the trend analyzation to a given period of only 10 years, from 2006 to 2016, which can be considered as the limitation of this study also. Besides, this study also undertook only Saudi Arabia resident population. The data, therefore, need to be further validated with recent data, when published in future to project the recent continuum of cancer prevalence and trends in the region, and can be extended to cover other population groups within the region to obtain a holistic cancer prevalence scenario.

## Conclusion

The current study highlights the trends and distribution of prevalence of esophageal cancer in the Saudi Arabian region. This study presents a clear and vivid picture of the prevalence of esophageal cancer based on gender, age groups, and regional distribution of the cases. There is an erratic but a gradual decrease in the trend of CIRs for esophageal cancer cases among men and women in Saudi Arabia. Also, ASIR for esophageal cancer incidences show a dynamic but retreating trend. The maximum mean ASIR for esophageal cancer in men and in women from 2006 to 2016 were found in the North Western region of Tabuk, while the Jazan region located in the southernmost part of Saudi Arabia along with the Northern region showcases minimum mean ASIRs in men and women, respectively. In the Northern region of Saudi Arabia, the maximum CIR and ASIR sex ratio was observed.

The study also highlights the importance of nutritional regime and lifestyle factors in cancer prevention and control with a special reference to esophageal cancer. Further studies are required to understand the roles of potential factors of esophageal cancer among the Saudi Arabia population to aid in cancer prevention and mitigation interventions in the future.

## Data Availability Statement

The original contributions presented in the study are included in the article/supplementary material, further inquiries can be directed to the corresponding author.

## Author Contributions

The author confirms being the sole contributor of this work and has approved it for publication.

## Conflict of Interest

The author declares that the research was conducted in the absence of any commercial or financial relationships that could be construed as a potential conflict of interest.

## Publisher's Note

All claims expressed in this article are solely those of the authors and do not necessarily represent those of their affiliated organizations, or those of the publisher, the editors and the reviewers. Any product that may be evaluated in this article, or claim that may be made by its manufacturer, is not guaranteed or endorsed by the publisher.
